# Increased nitric oxide availability attenuates high fat diet metabolic alterations and gene expression associated with insulin resistance

**DOI:** 10.1186/1475-2840-10-68

**Published:** 2011-07-22

**Authors:** Urszula Razny, Beata Kiec-Wilk, Lukasz Wator, Anna Polus, Grzegorz Dyduch, Bogdan Solnica, Maciej Malecki, Romana Tomaszewska, John P Cooke, Aldona Dembinska-Kiec

**Affiliations:** 1 Department of Clinical Biochemistry, Jagiellonian University Medical College, Kopernika 15a Street, 31-501 Cracow, Poland; 2 Department of Pathomorphology, Jagiellonian University Medical College, Grzegorzecka 16 Street, 31-531 Cracow, Poland; 3 Department of Metabolic Diseases, Jagiellonian University Medical College, Kopernika 15 Street, 31-501 Cracow, Poland; 4 Stanford University School of Medicine, 300 Pasteur Dr., Stanford, CA 94305-5406, USA

**Keywords:** eNOS-/- mice, DDAH mice, microarray, adipogenesis, angiogenesis

## Abstract

**Background:**

High fat diet impairs nitric oxide (NO) bioavailability, and induces insulin resistance. The link between NO availability and the metabolic adaptation to a high fat diet is not well characterized. The purpose of this study was to investigate the effect of high fat diet on metabolism in mice with decreased (eNOS-/-) and increased (DDAH overexpressed) NO bioavailability.

**Methods:**

eNOS-/- (n = 16), DDAH (n = 24), and WT (n = 19) mice were fed a high fat diet (HFD) for 13 weeks. Body weight, biochemical parameters, adipokines and insulin were monitored. The matrigel *in vivo *model with CD31 immunostaining was used to assess angiogenesis.

Gene expression in adipose tissues was analyzed by microarray and Real Time PCR. Comparisons of the mean values were made using the unpaired Student t test and p < 0.05 were considered statistically significant.

**Results:**

eNOS-/- mice gained less weight than control WT and DDAH mice. In DDAH mice, a greater increase in serum adiponectin and a lesser increment in glucose level was observed. Fasting insulin and cholesterol levels remained unchanged. The angiogenic response was increased in DDAH mice. In adipose tissue of DDAH mice, genes characteristic of differentiated adipocytes were down-regulated, whereas in eNOS-/- mice, genes associated with adipogenesis, fatty acid and triglyceride synthesis were upregulated.

**Conclusions:**

Our results indicate that increased NO availability attenuates some HFD induced alterations in metabolism and gene expression associated with insulin resistance.

## Background

Nitric oxide synthase (NOS) metabolizes L-arginine to L-citrulline and nitric oxide (NO), a key regulator of vascular and metabolic homeostasis. In the vasculature, the endothelial isoform (eNOS) exerts significant control over vessel tone, structure and interaction with circulating blood elements. Endothelium-derived NO is a potent vasodilator, diffusing into the underlying vascular smooth muscle to activate soluble guanylate cyclase, producing the second messenger cGMP [1]. Furthermore, NO is an angiogenic agent. Endothelial cell survival, proliferation, and migration are required for angiogenesis, and are promoted by NO [2]. As a signaling molecule low concentrations of NO (10-150 nmol/l) play a physiological role as an intra- and intercellular messenger [3]. For example, NO regulates metabolic lipid and carbohydrate metabolism [4,5]. Glucose metabolism is enhanced by NO, in part by upregulation of the Glut transporter, and possibly by enhanced vascular delivery of glucose to insulin sensitive tissues [6,7]. The importance of NO in vascular and metabolic homeostasis is highlighted by the observation that eNOS deficient mice have lower NO level [8], are hypertensive and insulin resistant [9,10]. A decreased production of NO by the mitochondrial form of NOS (mtNOS) has been proposed as a cause of decreased mitochondrial biogenesis, resulting in impairment of cellular turnover, tissue regeneration (heart, brain, liver) and aging [11,12]. On the other hand at high concentrations (>300 nmol/l) NO behaves as the cytotoxic molecule promoting the generation of hydroxyl radicals (HO*) [13].

Asymmetric dimethylarginine (ADMA) is an arginine analogue that acts as an endogenous inhibitor of the NOS pathway [14]. The enzyme dimethylarginine dimethylaminohydrolase (DDAH) degrades ADMA to citrulline and dimethylamine, and exists as two isoforms (DDAH-1 and -2) [15,16]. Whereas deficiency of either isoform is lethal, the heterozygous deficient animals manifest increased plasma levels of ADMA, synthesize less NO, and are hypertensive [17]. By contrast, mice that over express DDAH-1 have lower ADMA levels, greater NOS activity and in consequence higher NO levels [18] and lower blood pressure. Intriguingly, these mice are also insulin sensitive [19].

A HFD is known to impair NO stability and synthesis, and to induce insulin resistance. We were interested to know if differing basal capacities to generate NO would affect the metabolic adaptation to a HFD. Accordingly, we studied the response to a HFD of normal C57Bl6J mice; those that were deficient in NO synthesis (eNOS-/-); and those that had enhanced NO synthesis (DDAH-1 overexpression).

## Methods

### Mice

The eNOS deficient animals eNOS-/- (B6.129P2-Nos3tm1Unc/J) were purchased from Jackson Laboratory (USA), and transgenic DDAH mice C57BL/6J-TG (ACTB-DDAH1)1Jpck/J from Charles River Laboratories (Sulzfeld, Germany).

The eNOS-/- transgenic mice lack endothelial nitric oxide synthase activity. The mice were created using a construct that replaced 129 bp of exon 12 of the Nos3 (eNOS) gene with a 1.2 kb neomycin cassette so as to disrupt calmodulin binding [10].

The transgenic DDAH mice were offspring of control females and DDAH transgenic males overexpressing dimethylarginine dimethylaminohydrolase. The mice were created using a construct encoding human DDAH I cDNA, a human B-actin promoter, and RNA processing signals from SV40 derived from a modified human agouti expression vector [15], called in this work "DDAH mice". The C57BL/6J mice are the background strain for DDAH as well as the eNOS-/- animals and they served as control animals.

Animals were housed in cages (22°C and 12 hour daylight cycle) and were fed standard rodent feed (9 en% of fat) (Gam-rat, Lublin, Poland). At the age of 6 weeks, C57BL/6J (n = 19), eNOS-/- (n = 16) and DDAH (n = 24) female mice were entered into the experimental protocol and fed a high saturated fat diet (coconut oil hydrogenated based, 39 en% of fat) (MP Biomedicals, Solon, Ohio, USA) for 13 weeks ad libitum. During that period we monitored body weight (3 times weekly), feed consumption (once weekly), and blood biochemical parameters (every 2 weeks).

This study was conducted according to National Institutes of Health Guide for the Care and Use of Laboratory Animals and was approved by the Institutional Animal Care Committee. All experimental protocols and procedures were approved by the Local University Ethic Committee in Cracow (No 83/OP/2005) and performed in accordance with the policies regarding the human care and use of laboratory animals. The performed research was in compliance with ARRIVE guidelines on animal research [20].

### Biochemical parameters

The serum glucose, triglycerides and total cholesterol concentrations were measured (Cormay Diagnostic Kit; Lublin, Poland) in blood samples collected from the tail vein, after 4 hours of fasting. In addition serum concentrations of insulin, leptin and adiponectin concentrations were measured at age 6 and 19 weeks by ELISA (Linco Research, St. Charles, Missouri, USA or R&D Systems, McKinley Place NE Minneapolis, USA) [21]. Intra- and interassay coefficients of variation (CV) were 8,35 and 17,9% for insulin, 4,3 and 7,6% for leptin, 6,6 and 6,4% for adiponectin.

In order to assess insulin sensitivity, the following indices were calculated: homeostasis model assessment (HOMA) and quantitative insulin sensitivity check index (QUICKI). Both indices are based on the fact that the relationship between glucose and insulin in the basal state reflects the balance between hepatic glucose output and insulin secretion. QUICKI and HOMA indexes were calculated as described previously [22]: QUICKI = 1/[log(I) + log(G)], where I is fasting insulin (mU/l) and G is fasting glucose (mg/dl).; HOMA = G * I/22.5, where I is fasting insulin (mU/l) and G is fasting glucose (mmol/l).

### Subcutaneous matrigel model of angiogenesis

Six days before the end of the feeding period, mice received subcutaneous injections of 0.5 ml matrigel (Becton Dickinson, Franklin Lakes, New Jersey, USA) containing basic fibroblast growth factor (bFGF, 25 nmol/l final concentration) (Sigma-Aldrich, St. Louis, Missouri, USA) [23]. After 6 days mice were killed and matrigel plugs were excised with surrounding tissue and preserved for immunohistochemical staining with rat anti-mouse CD31 (PECAM1) antibody (Becton Dickinson, Franklin Lakes, New Jersey, USA) [24]. Angiogenic response was estimated by the amount of PECAM1-positive structures and expressed as the number of vessels with or without a lumen as well as number of individual PECAM1-positive cells counted with a Hot Spots method (5 frames per slide and 3 slides per plug) by an experienced histopathologist that was blinded to the experimental groups.

### Collection of adipose tissue

After mice were killed samples of inguinal white (WAT) and brown adipose tissue from the interscapular area (BAT) were preserved in Trizol Reagent (Invitrogen, Carlsbad, California, USA) for gene expression analysis.

### Analysis of gene expression

For gene expression analysis mRNA was isolated from white or brown adipose tissues using Trizol Reagent (Invitrogen, Carlsbad, California, USA) and purified with QIAamp RNA for total RNA isolation system (Qiagen, Valencia, California, USA). The quality of RNA was confirmed by denaturing gel electrophoresis and an analysis on the Agilent 2100 Bioanalyser (Agilent Technologies, Foster City, California, USA). High grade RNA was used for hybridization with NuGO oligonucleotide microarrays (NuGO_Mm1a520177) designed by NuGO and manufactured by Affimetrix (Fremont, California, USA). The microarray assay was used to evaluate the effects of impaired (mice eNOS-/-) and enhanced (DDAH -1 overexpressing mice) NO synthase activity on genes involved in the metabolism of white or brown adipose tissue. Comparison of relative gene expression for eNOS-/- versus DDAH mice (white and brown adipose tissues separately) were calculated using GCOS 1.4 software (Affymetrix, Fremont, California, USA). Results from the microarray were presented as relative gene expression values (fold change). Only genes for which expression was significantly regulated more than 1.4 fold were analyzed further.

Most of the significantly regulated genes related to angiogenesis, adipogenesis, fatty acid synthesis, nuclear receptors in lipid/glucose metabolism and cytotoxicity. These findings were confirmed by quantitative real-time PCR (qRT-PCR). *Gapdh *was used as a reference gene.

### Statistical Analysis

Results are shown as mean value ± standard deviation (SD). Changes in the serum levels of cytokines and adipokines are presented as Δ value between the initiation of the dietary intervention (at 6 weeks) and sacrifice of the animals (19 weeks). Comparisons of the mean values were made using the unpaired Student t test and p < 0.05 were considered statistically significant.

Microarrays were analyzed with Affymetrix Microarray Analysis Suite. Changes in relative gene expression were calculated as a rate of case strain (eNOS-/- or DDAH) against controls (C57/BL/6J) using GeneChip Operating Software (GCOS 1.4). Only genes with significant differences in signal intensity of at least 1.4 fold and p < 0.05 were included for further analysis. Analysis of regulated pathways was performed using Genemap software.

## Results

### Body composition, biochemical parameters

**Figure 1:**
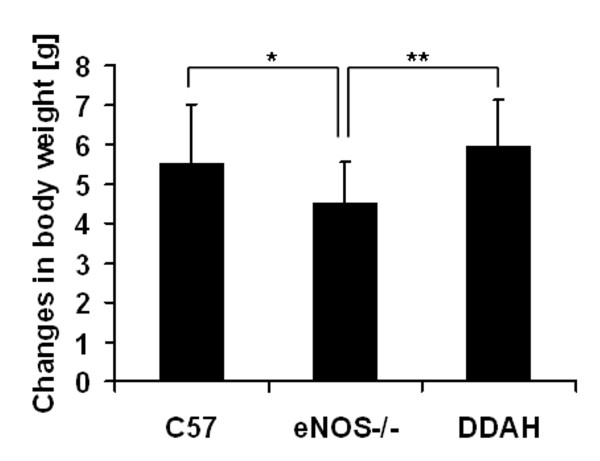
**Changes in body weight**. Control C57BL/6J (n = 19), eNOS-/- (n = 16) and DDAH (n = 24) mice were fed with high fat diet during 13 weeks. Values are expressed as means ± SEM; significance *p < 0.05; **p < 0.01.

**Figure 2:**
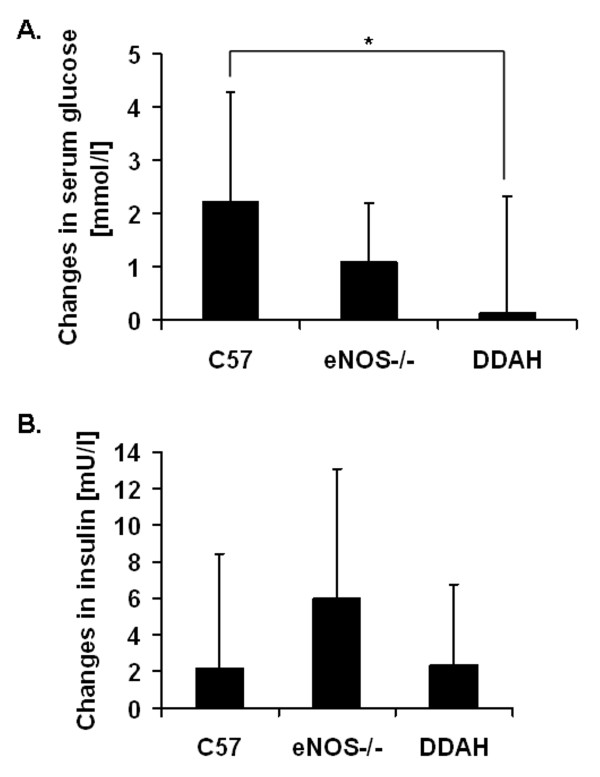
**Changes of serum glucose (A) and insulin levels (B)**. The C57BL/6J (n = 19), eNOS-/- (n = 16), DDAH (n = 24) animals were fed a high fat diet for 13 weeks. Values are expressed as means ± SEM; significance *p < 0.05.

Body mass measurements revealed that eNOS deficient mice gained less weight by comparison to control C57 (WT) and DDAH mice (Figure 1). The 13 weeks of the high fat diet (HFD) was associated with an increase in blood serum glucose by over 2 mmol/l in the control mice (Figure 2A). A smaller increment was observed in eNOS-/- mice while in the DDAH group there was almost no increase in glucose concentration (Figure 2A). Differences between DDAH and control mice were statistically significant. The high fat diet caused similar elevations in serum cholesterol levels (by 1.2 - 1.7 mmol/l) in each group. Triglyceride levels fluctuated during the feeding period and there were no differences between mouse strains (data not shown).

**Figure 3:**
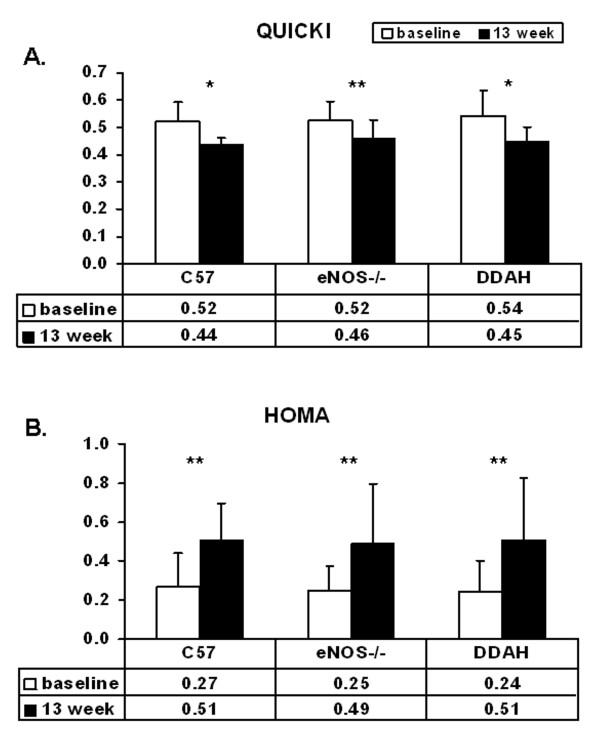
**Insulin sensitivity of C57BL/6J (n = 19), eNOS-/- (n = 16) and DDAH (n = 24) mice**. Insulin sensitivity was shown at baseline and after 13 weeks of feeding with a high saturated fat diet. Values are expressed as means ± SEM, *p < 0.05; **p < 0.01, significance between baseline and 13 weeks of feeding with a high fat diet. **(A) **Quantitative insulin sensitivity check index (QUICKI) (baseline and after 13 weeks of high fat diet feeding). QUICKI = 1/[log(I) + log(G), where I is fasting insulin (mU/l) and G is fasting glucose (mg/dl). **(B) **Homeostasis model assessment (HOMA) (baseline and after 13 weeks of high fat diet feeding). HOMA = G * I/22,5, where I is fasting insulin (mU/l) and G is fasting glucose (mmol/l).

There were no significant differences between groups in insulin levels before or after dietary intervention (Figure 2B) (Table 1). The high fat diet caused similar increases in measures of insulin resistance in each strain. The high fat diet induced a reduction in the QUICKI (Figure 3A) and an increase in the HOMA indices (Figure 3B) in all groups, consistent with dietary induced insulin resistance (Table 1).

**Table 1:** Comparison of biochemical parameters amongst C57, eNOS-/- and DDAH mice before (baseline) and after feeding a high fat diet (week 13).

	C57		eNOS-/-		DDAH	
	
	baseline	13 week	baseline	13 week	baseline	13 week
**Glucose [mmol/l]**	8.34 ± 2.20	9.91 ± 1.31*	8.80 ± 1.07	9.89 ± 1.15	9.14 ± 1.94	9.28 ± 1.92*

**Insulin [mU/l]**	7.91 ± 5.04	10.07 ± 4.32	5.52 ± 2.40	11.51 ± 6.24**	6.24 ± 3.84	10.55 ± 5.52*

**QUICKI^a^**	0.52 ± 0.07	0.44 ± 0.02*	0.52 ± 0.07	0.46 ± 0.07**	0.54 ± 0.09	0.45 ± 0.05*

**HOMA^b^**	0.27 ± 0.17	0.51 ± 0.19**	0.25 ± 0.13	0.49 ± 0.31**	0.24 ± 0.16	0.51 ± 0.32**

^a^**QUICKI = 1/[log(I) + log(G), where I is fasting insulin (mU/l) and G is fasting glucose (mg/dl)**

^b^HOMA = G * I/22.5, where I is fasting insulin (mU/l) and G is fasting glucose (mmol/l)

*p < 0.05; **p < 0.01: significance between baseline and after13 weeks of feeding (week 13)

**Figure 4:**
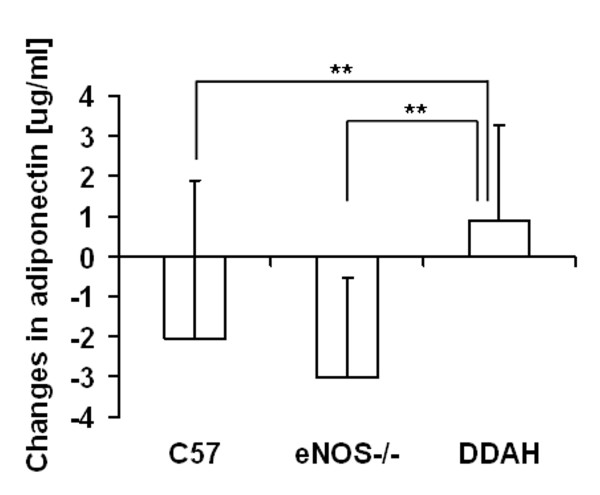
**Changes in serum adiponectin concentration**. The C57BL/6J (n = 19), eNOS-/- (n = 16), DDAH (n = 24) animals were fed a high fat diet for 13 weeks. Values are expressed as means ± SEM; significance *p < 0.05, **p < 0.01.

Nevertheless, serum levels of adipokines revealed some intriguing group differences. In the DDAH transgenic mice, we observed a greater increase in serum adiponectin levels by comparison to control and eNOS-/- mice. The levels of this adipokine decreased during the feeding period from 9.7 μg/ml to 7.5 μg/ml in control mice and from 9.6 μg/ml to 6.6 μg/ml in eNOS-/- mice (Figure 4). No group differences in leptin levels were observed before or after the dietary intervention.

### Angiogenic response in matrigel plug

**Figure 5:**
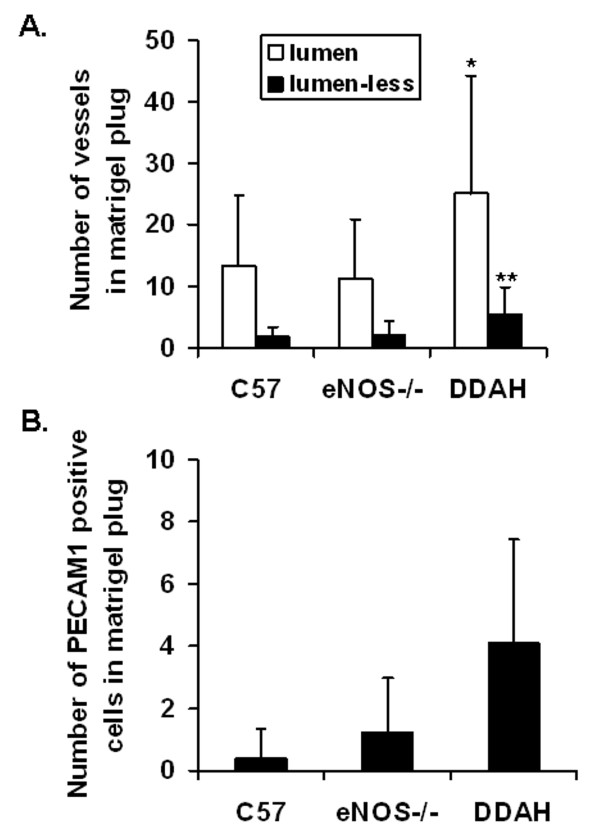
**Angiogenic response in matrigel plug from C57BL/6J (n = 19), eNOS-/- (n = 16), DDAH (n = 24) mice**. Values are expressed as means ± SEM; significant increase in DDAH compared to control (C57) and eNOS-/- mice *p < 0.05, **p < 0.01. **(A) **The number of CD31 (PECAM1) positive vessel like structures in matrigel plug of control C57BL/6J (n = 19), eNOS-/- (n = 16) and DDAH (n = 24) mice. **(B) **The number of single CD31 (PECAM1) positive cells present in matrigel plug of C57BL/6J (n = 19), eNOS-/- (n = 16) and DDAH (n = 24) mice.

The angiogenic response was enhanced in the DDAH animals. In the subcutaneous matrigel plugs, the number of vessels with a lumen, the number of vessels without a lumen as well as the number of single PECAM1 positive cells was significantly higher in DDAH transgenic mice by comparison to control or eNOS-/- mice. There were no differences in the angiogenic response to matrigel between eNOS deficient mice and controls (Figure 5).

### Effects of HFD on adipose gene expression

Morphology of adipocytes in adipose tissue from eNOS knockout mice and DDAH mice did not differ. The influence of eNOS deletion and DDAH overexpression on gene expression in brown (BAT) or white (WAT) adipose tissue are presented in Table 2, 3, 4, 5, 6.

**Table 2:** Relative changes in gene expression connected with adipogenesis for white (WAT) and brown (BAT) adipose tissues of eNOS -/- and DDAH animals vs. control mice.

		WAT		BAT	
		**eNOS-/-**	**DDAH**	**eNOS-/-**	**DDAH**

**Adipogenesis**					

CCAAT/enhancer binding protein (C/EBP)	*Cebpa*	↑			**↓**

	*Cebpb*	↑			**↓**

	*Cabpd*			↑	

peroxisome proliferator activated receptor alpha	*Ppara*		**↓**		**↓**

peroxisome proliferator-activated receptor gamma	*Pparg*		**↓#**		

peroxisome proliferator-activated receptor beta	*Pparb*	↑			

retinoid X receptor gamma	*Rxrg*		**↓**		

forkhead box O	*Foxo1*	↑	↑		**↓**

	*Foxo3a*				**↓**

myocyte enhancer factor	*Mef2c*		**↓**		

	*Mef2d*	↑			**↓**

nuclear receptor subfamily 2, group F, member 1	*Nr2f1*	↑			

nuclear receptor interacting protein	*Nrip*	**↓**	**↓**		↑

nuclear receptor co-repressor	*Ncor*	↑			

transforming growth factor, beta	*Tgfb*	↑			

insulin-like growth factor 1	*Igf1*				↑

Kruppel-like factor 6	*Klf6*		**↓**	↑	

Kruppel-like factor 7	*Klf7*			↑	

nuclear receptor subfamily 3, group C, member 1	*Nr3C1*		**↓**		

prolactin receptor	*Prlr*	**↓**			

early B-cell factor 1	*Ebf1*	**↓**			

growth differentiation factor 10	*Gdf10*	↑	**↓**		

catenin (cadherin associated protein)	*Catnb*		↑		↑

early growth response	*Egr*		↑		

zinc finger, HIT type 3	*Trip3*				↑

stearoyl-Coenzyme A desaturase 1	*Scd1*		↑		

inhibitor of DNA binding 3	*Id3*			↑	

phosphoenolpyruvate carboxykinase 1, cytosolic	*Pck1*				↑

interleukin 6 signal transducer	*Il6st*				**↓**

**Adipocyte differentiation markers**					

fatty acid binding protein 4	*Fabp4*	**↓**	**↓#**	↑	

uncoupling protein 1	*Ucp1*	↑**#**	**↓#**		**↓#**

lipoprotein lipase	*LPL*		**↓#**		**↓#**

lipase, hormone sensitive	*Lipe*		**↓**	**↓**	

facilitated glucose transporter	*Glut4*	↑**#**		**↓#**	

**Adipocyte secretion products**					

angiotensinogen	*Agt*	**↓**	**↓**		

adipsin	*Adn*				**↓**

adipophilin	*Adfp*				**↓**

↑ - upregulated genes; ↓ - downregulated genes; # - genes which expression was confirmed by real time PCR

**Table 3:** Relative changes in gene expression related to lipid metabolism for white (WAT) and brown (BAT) adipose tissues of eNOS -/- and DDAH animals vs. control mice.

		WAT		BAT	
		**eNOS-/-**	**DDAH**	**eNOS-/-**	**DDAH**

**Lipodystrophy related genes**					

lipin	*Lipin1*	↑			**↓**

lysophosphatidic acid acyltransferase, beta	*Agpat2*	↑			**↓**

zinc metallopeptidase	*Zmpste24*		**↓**		

Bernardinelli-Seip congenital lipodystrophy	*Bscl2*				**↓**

**Cholesterol synthesis**					

hydroxymethylglutaryl-CoA synthase 1	*Hmgcs1*	↑	↑		

farnesyl diphosphate synthetase	*Fdps*		↑	**↓**	

phosphomevalonate kinase	*Pmvk*				**↓**

mevalonate kinase	*Mvk*	↑	↑		

squalene epoxidase	*Sqle*			**↓**	**↓**

7-dehydrocholesterol reductase	*Dhcr7*	↑	↑		**↓**

lanosterol synthase	*Lss*		↑		

cytochrome P450, family 51	*Cyp51*		↑		

sterol-C5-desaturase	*Sc5d*		↑		

**Triglyceride synthesis**					

diacylglycerol O-acyltransferase	*Dgat1*		**↓**		

	*Dgat2*	↑			

lysophosphatidic acid acyltransferase, beta	*Agpat2*	↑			

**Fatty acid synthesis**					

fatty acid synthase	*Fasn*	↑**#**	**↓#**		**↓#**

stearoyl-Coenzyme A desaturase	*Scd*		↑		

ATP citrate lyase	*Acly*	↑		**↓**	

acetyl-Coenzyme A carboxylase beta	*Acacb*	**↓**	**↓**	**↓**	**↓**

mitochondrial trans-2-enoyl-CoA reductase	*Nrbf1*	**↓**			

enoyl coenzyme A hydratase 1	*Ech1*				**↓**

**Fatty acid beta-oxydation**					

patatin-like phospholipase domain containing 2	*Pnpla2*	**↓**			

mitochondrial trifunctional protein, alpha subunit	*Hadha*		**↓**		**↓**

fatty acid Coenzyme A ligase	*Acsl1*			**↓**	

	*Acsl3*				**↓**

	*Acsl5*	**↓**			

carnitine palmitoyltransferase 1a	*Cpt1a*		↑		

acyl-Coenzyme A dehydrogenase	*Acads*				**↓**

	*Acadvl*				**↓**

mitochondrial carnitine/acylcarnitine translocase	*Slc25a20*				**↓**

triosephosphate isomerase 1	*Tpi1*				**↓**

glycerol kinase	*Gyk*				**↓**

carnitine acetyltransferase	*Crat*				**↓**

carnitine palmitoyltransferase 2	*Cpt2*				**↓**

carnitine palmitoyltransferase 1b	*Cpt1b*				**↓**

↑ - upregulated genes; ↓ -downregulated genes; # - genes which expression was confirmed by real time PCR

**Table 4:** Relative changes in gene expression related to carbohydrate metabolism for white (WAT) and brown (BAT) adipose tissues of eNOS -/- and DDAH animals vs. control mice.

		WAT		BAT	
		**eNOS-/-**	**DDAH**	**eNOS-/-**	**DDAH**

**Insulin signaling**					

insulin receptor substrate	*Irs1*			↑	

	*Is3*				**↓**

growth factor receptor bound protein 2-associated protein 1	*Gab1*				**↓**

phosphatidylinositol 3-kinase	*PIk3r1*	**↓**	**↓**		↑

	*PIk3r2*	↑			

	*PIk3ca*		**↓**		

phosphatase and tensin homolog	*Pten*	**↓**	**↓**		

integrin linked kinase	*Ilk*	↑			

actinin	*Actn*	↑			

vinculin	*Vcl*		↑		

filamin	*Flna*	↑	**↓**		

zyxin	*Zyx*	↑			

vasodilator-stimulated phosphoprotein	*Vasp*	↑			

RAS-related C3 botulinum substrate 1	*Rac1*		↑		

**AKT/PDK**					

thymoma viral proto-oncogene 2	*Akt2*	↑			

serum/glucocorticoid regulated kinase	*Sgk*		**↓**	↑	

glycogen synthase kinase 3 beta	*Gsk3b*	↑			**↓**

**m-TOR**					

tuberous sclerosis	*Tsc1*				**↓**

	*Tsc2*	↑			

mechanistic target of rapamycin (serine/threonine kinase)	*frap1*				**↓**

ribosomal protein S6 kinase	*Rps6kb1*	↑			↑

	*Rps6kb2*	**↓**			

eukaryotic translation initiation factor 4E binding protein	*Eif4ebp1*				**↓**

**Glycolysis/Gluconeogenesis**					

enolase	*Eno3*	↑**#**			**↓**

glucose phosphate isomerase 1	*Gpi1*	↑			**↓**

aldolase	*Aldoa*	↑			**↓**

↑ - upregulated genes; ↓ -downregulated genes; # - genes which expression was confirmed by real time PCR

**Table 5:** Relative changes in gene expression connected with oxidative stress and inhibition of angiogenesis for white (WAT) and brown (BAT) adipose tissues of eNOS -/- and DDAH animals vs. control mice.

		WAT		BAT	
		**eNOS-/-**	**DDAH**	**eNOS-/-**	**DDAH**

**Anti-Angiogenic genes**					

**Adhesion**					

thrombospondin 1	*Thbs1*	**↓**			

thrombospondin 2	*Thbs2*	**↓**	**↓**		

Collagen		↑7subunits	↑3subunits		

			**↓**1 subunit		

laminin B1	*Lamb1-1*				↑

Laminin alpha	*Lama*		↑		

Laminin beta	*Lamb*		↑		

integrin alpha	*Itga6*		**↓**	↑	

	*Itga7*	↑			

	*Itga8*	↑	**↓**		

	*Itgam*	**↓**	↑		

**MMP's**					

tissue inhibitor of metalloproteinase	*Timp3*		**↓**		

	*Timp4*	**↓**	**↓**	**↓**	

**Apoptosis**					

nuclear factor of kappa B	*Nfkbia*	**↓**	**↓**		

caspase	*Casp7*				**↓**

	*Casp8*				↑

BCL2-associated X protein	*Bax*	**↓**			

conserved helix-loop-helix ubiquitous kinase	*Chuk*		↑		

transformation related protein 53	*Trp53*	↑			

BCL2-related ovarian killer protein	*Bok*	↑			

X-linked inhibitor of apoptosis			↑		↑

**Oxidative stress**					

**Repression of ROS production**					

cytochrome b-245, alpha polypeptide	*Cyba*			↑	↑

nuclear factor I/X	*Nfix*			**↓**	

glutathione S-transferase, theta 2	*Gstt2*	**↓**			

Gtpase activating protein	*Gyp1a1*	**↓**			

monoamine oxidase A	*Maoa*	**↓**			

**Adaptation against existing ROS**					

FBJ osteosarcoma oncogene	*Fos*		↑	↑	↑

superoxide dismutase 2	*Sod*		↑	**↓**	**↓**

glutathione S-transferase theta	*Gstt*		↑		

glutathione peroxidase 3	*Gpx*				**↓**

catalase	*Cat*				**↓**

thioredoxin 2	*Txn2*				**↓**

↑ - upregulated genes; ↓ -downregulated genes; # - genes which expression was confirmed by real time PCR

**Table 6:** Relative changes in pro-angiogenic gene expression for white (WAT) and brown (BAT) adipose tissues of eNOS -/- and DDAH animals vs. control mice.

		WAT		BAT	
		**eNOS-/-**	**DDAH**	**eNOS-/-**	**DDAH**

**Pro - Angiogenic genes**					

**MMP's**					

Stromielins		**↓**	**↓**		

basigin	*Bsg*				↑

Gelatinases			**↓**		

matrix metallopeptidase	*Mmp19*	↑			

	*Mmp17*	↑			

**Proliferation**					

Rho-associated coiled-coil containing protein kinase 1	*Rock1*	↑			

coatomer protein complex, subunit beta	*Rack2*		**↓**		

myosin, light polypeptide kinase	*Mylk*	↑	**↓**		

proliferating cell nuclear antigen	*Pcna*				↑

macrophage expressed gene 1	*Mpeg1*				↑

cyclin H	*CCnh*				↑

Jun oncogene	*Jun*			↑	**↓**

FBJ osteosarcoma oncogene	*Fos*			↑	↑

**Cell survival**					

RAF proteins		↑	**↓**	↑	

ERK proteins		↑			

X-linked inhibitor of apoptosis	*Birc4*		↑		

Jun oncogene	*Jun*			↑	**↓**

**Apoptosis**				↑	

heat shock protein	*Hspa1a*			↑	

**Adhesion**					

platelet derived growth factor receptor, alpha	*Pdgfra*			↑	

fibronectin	*Fn1*	↑			

tenascin XB	*Tnxb*	**↓**	**↓**		

von Willebrand factor	*Vwf*		**↓**		

↑ - upregulated genes; ↓ -downregulated genes; # - genes which expression was confirmed by real time PCR

#### Pro-adipogenic genes

We observed distinctly different responses in gene expression in response to high fat feeding (Table 2). In the eNOS knockout (ko) mice, the expression of proadipogenic (and the adipocyte differentiation markers) genes were increased in WAT (*Cebpa, Cebpb, Ppadb, Foxo1, Nr2f1, Mef2d, Ncor, Tgfb, Gdf10, Ucp1, Glut4), *and BAT (*Cebpd, Klf6, Klf7, Id3, Fabp)*. By contrast, in the DDAH transgenic animals, there was primarily downregulation of adipogenic gene expression in WAT (*Ppara, Pparb, Rxrg, Mef2c, Nrip,Klf6, Nr3c1, Gdf10, Fabp4, Ucp1, Lipe, Agt), *and in BAT (*Cebpa, Cebpb, Ppara, Foxo1, Foxo3a, Mef2d, LPL, Il6st)*. The proadipogenic genes *Cebpa, Cebpb, Foxo1, Mef2d, Ucp1, Gdf10 *were also differentially regulated in BAT and WAT in eNOS ko (up) versus DDAH transgenic (down) animals. Lipodystrophy related genes were also induced in WAT of eNOS ko animals (*Lipin1, Agpat2).*

#### Lipid biosynthesis genes

Genes related to fatty acid synthesis (*Fasn, Acly) *were up-regulated in WAT of eNOS ko animals, while most of such genes (*Fasn, Acacb, Ech1*) were primarily down-regulated in DDAH animals (Table 3). The fatty acid synthase gene (*Fasn*) was differently expressed in the animal models (upregulated in eNOS ko, while downregulated in the DDAH mice). Triglyceride biosynthesis related genes were also upregulated in WAT of eNOS ko animals (*Dgat2, Agpat2*). HFD increased the cholesterol biosynthesis genes in WAT of eNOS ko (*Hmgcs1, Mvk, Dhcr7*) and the DDAH mice (*Hmgcs1, Fdps, Mvk, Dhcr7, Lss, Cyp51, Sc5d). *By contrast, several cholesterol biosynthesis genes (*Pmvk, Sqle, Dhcrt) *were downregulated in BAT of DDAH mice.

#### Genes involved in lipid and carbohydrate metabolism

By comparison to the control animals, we observed downregulation of the expression of genes related to beta-oxidation of fatty acids in the DDAH mice (*Hadha, Acads, Acadvl, Slc25a20, Tpi1, Gyk, Crat, Cpt2,Cpt1b*) (Table 3).

The insulin-signaling related genes (were upregulated in WAT of eNOS ko animals (*Plk3r2, Ilk, Actn, Flna, Zyx, Vasp, Akt2, Gsk3b)*, while downregulated in the DDAH animals (*Plk3r1, Plk3ca, Pten, Flna, Sgk) *(Table 4).

Long-term HFD feeding resulted in the upregulation of genes related to glycolysis/gluconeogenesis (*Eno3, Gpi1, Aldoa) *in WAT of eNOS ko, whereas some of these were downregulated in BAT of DDAH animals (Table 4).

#### Genes related to oxidative stress and angiogenesis

Genes protective against oxidative stress were downregulated in WAT of eNOS ko, (*Gstt2,Gyp1a1, Maoa) *while up-regulated (*Fos, Sod, Gstt*) in the DDAH animals. Downregulation of such genes (*Sod, Gpx,Cat, Txn2*) was primarily observed in BAT of DDAH mice (Table 5).

We observed upregulation of some adhesion and cell survival/proliferation-related genes (in WAT (*Lama, Lamb, Itgam, Birc4*, *x-linked inhibitor of apoptosis) *of the DDAH mice as well as in the eNOS ko animals (*Fn1, Itga7, Itga8, Mmp19, Mmp17, Mylk, Trp53, Bok, RAF, ERF*). Downregulation of some angiogenic genes in WAT of eNOS and DDAH animals (*Thbs2,Tnxb, stromelins, Timp4, Nfkbia, vWF*) was also observed (Table 5, 6). By comparison to WAT, in BAT tissue the angiogenic genes were less regulated; however, some genes for proliferation and antiapoptotic gene expression were upregulated in the DDAH animals (*Pcna, Mpeg1, CCnh, Fos, X-linked inhibitor of apoptosis*).

## Discussion

In metabolic disorders associated with atherosclerosis, NO synthesis and/or stability is reduced [5,6,14]. To determine if NO bioavailability may modulate the response to a high fat diet, we assessed serum and genetic markers of metabolism in mice with decreased (eNOS ko) as well as increased (DDAH) NO bioavailability.

We found that differing basal levels of NO synthetic capacity influence the response to a HFD as assessed by glucose and adiponectin levels; the angiogenic response; and adipose (BAT and WAT) gene expression. The data suggest that in aggregate, NO activity is protective against some of the metabolic perturbations induced by a high fat diet.

### Diet-induced insulin resistance

Epidemiological, clinical and basic research studies have demonstrated that a high fat diet induces insulin resistance. Most studies suggest that increased dietary fat causes whole-body and regional (liver, adipose tissue, muscle) insulin resistance in both animals and humans. Vessby et al. documented that insulin sensitivity was impaired by 10% in healthy individuals who receive an isoenergetic diet containing a high content of saturated fatty acids for 3 months. A change in the composition of the dietary fatty acids, ie. decreasing saturated fatty acid and increasing monounsaturated fatty acid content, improved insulin sensitivity [25]. Substituting 11% of the saturated fatty acids with short-chain omega-3 fatty acids (alpha-linolenic acid, 18:3 omega 3) prevented insulin resistance induced by a saturated-fat diet in rats [26].

Duplain [9] and Sydow [19] using glucose clamp studies reported insulin resistance in eNOS ko mice and increased insulin sensitivity in DDAH transgenic mice. It has been reported that elevated plasma levels of ADMA are associated with insulin resistance, micro/macrovascular diabetic complications, and may predict cardiovascular events in type 2 diabetic patients. In turn, Lu et al. documented that some genetic variations in DDAH1 could contribute to higher risk of type 2 diabetes independently of plasma ADMA levels.

For example, SNP (single nucleotide polymorphism) rs1241321 in DDAH1 was found to be associated with a higher type 2 diabetes risk independently of plasma ADMA levels. AA genotype at rs1241321 appeared to be more insulin-sensitive in comparison to AG/GG individuals. Thus, the DDAH1 gene could play an important role in the pathogenesis of type 2 diabetes [27].

In our studies in order to access insulin resistance we focused on the fasting levels of glucose and insulin. Our work confirms that a high fat diet rich in saturated fatty acids induces insulin resistance, which was observed in all groups. However, the DDAH animals were resistant to the diet-induced increase in glucose levels observed in the control animals. This was despite a greater weight gain in the DDAH transgenic animals in response to the high fat diet (see discussion below). Previous studies by Tanaka [18] demonstrated higher NO level in DDAH mice thus lower glucose levels in the DDAH transgenic mice reflect the fact that NO is known to increase glucose transport, in part by increasing the translocation to the cell surface of Glut 4, the active transporter of glucose [28]. In skeletal muscle from eNOS ko mice, which according to Kanetsuna studies present lower NO levels [8], there is diminished insulin-stimulated glucose uptake, indicating that insulin activation of NO may contribute to the stimulation of glucose transport [9].

Furthermore, the DDAH transgenic animals exhibited higher adiponectin levels. Adiponectin is an adipocytokine that increases glucose uptake, reduces gluconeogenesis and lipogenesis, and enhances β-oxidation of fat, by activating AMPK and PPARα [29]. A characteristic feature of individuals with diabetes mellitus or insulin resistance is a decrease of adiponectin levels [30]. There appears to be a reciprocal relationship between adiponectin and NO. Adiponectin deficient mice exhibit impaired endothelium-dependent vasodilation [31]. This is likely due to the fact that adiponectin increases the stability of eNOS mRNA and half-life, enhances the association of eNOS with Hsp90 and stimulates the phosphorylation of eNOS, which together lead to increased NO production [32,33]. Adiponectin may also prevent NO degradation by reducing the production of superoxide anion by endothelial cells [34]. On the other hand, NO appears to positively regulate adiponectin levels. Chronic administration of the NOS inhibitor L-NAME to Sprague-Dawley rats reduces plasma adiponectin levels [35]. Conversely, chronic enhancement of NO synthesis (the DDAH transgenic mouse) is associated with resistance to diet-induced reduction in adiponectin levels (the current study).

### High fat diet and angiogenesis

Endothelial NOS activity plays a critical role in angiogenesis and endothelial function. Survival, proliferation and migration of endothelial cells, and their secretion of angiogenic factors, are dependent upon NO [36,37]. The angiogenic response to ischemia after femoral artery ligation is impaired in eNOS deficient mice, whereas it is enhanced in eNOS overexpressing mice [38]. Similarly, after femoral artery ligation, chronic infusion of ADMA reduces capillary density and perfusion, whereas DDAH overexpression (associated with lower endogenous ADMA levels) increases angiogenic response and improves endothelial function [39].

One of the diseases associated with impaired endothelium-dependent NO mediated vasodilation (endothelial dysfunction) is congestive heart failure (CHF). Coronary endothelial dysfunction in CHF may be due to decreased DDAH, which in consequence, leads to a higher ADMA level [40]. Moreover, Riccioni et al. showed that patients who received pharmacological treatment for acute congestive HF (diuretics, digoxin, ACE-inhibitors or angiotensin receptor blockers, and nitroglicerin), have significantly higher plasma ADMA levels after pharmacological treatment in comparison to pre-treatment. Such results suggest that acute renal impairment function and the modulation of NOS determine plasma ADMA levels after therapy [41].

The current study concentrated on the effect of NO availability on angiogenesis in mice. To achieve this aim, another model of angiogenesis, in response to subcutaneous administration of matrigel, was used. Consistent with previous studies, we observed that the vascularity of the matrigel plug was increased in the DDAH transgenic mice, as manifested by a higher number of vessel-like structures (with and without lumen) as well as a greater number of PECAM1 positive cells. Since angiogenesis is crucial for development and growth of all organs (including adipose), NOS activity may in part explain the discordance in body weight gain with the HFD in the different groups. By comparison to the control mice, the HFD induced a greater weight gain in the DDAH mice, whereas in the eNOS-/- animals it induced less weight gain. This observation is consistent with a previous report that eNOS deficient female mice are smaller than wild type ones [42].

We were surprised to find that angiogenesis in response to the matrigel plug was not reduced in the eNOS-/- mice. It is possible that the matrigel itself, containing a number of angiogenic cytokines including bFGF [43] potently recruits other angiogenic mechanisms, including inflammatory cells carrying iNOS [44]. It is also possible that in the setting of a HFD, the concomitant inflammation and oxidative stress contribute to the angiogenic stimulus.

### Angiogenesis, lipogenesis and metabolism

Adipose tissue is highly vascularized, and each adipocyte is nourished by an extensive capillary network. Adipose tissue may be considered as the largest endocrine gland, as it produces several systemically active adipokines (eg. leptin, adiponectin, resistin) as well as VEGF, angiopoietins, HGF, IGF-1, angiogenin, IL-6, TNF-α and fatty acid metabolites. Many of these may promote the inflammation and angiogenesis response associated with adipose tissue accumulation [45]. It is also documented that adipose tissue endothelial cells promote preadipocyte differentiation [46]. Vascularity of adipose tissue is, therefore, critical for development, maturation, plasticity and functions of adipose tissue as a metabolic and an endocrine organ [47,48]. Thus, it may seem counterintuitive that, if angiogenesis promotes lipogenesis, then the DDAH animals (which have an enhanced angiogenic response) have a normalized metabolic state. However, DDAH overexpression may have additional effects to offset any metabolic effects of angiogenesis-enhanced lipogenesis. For example, the reduction in ADMA would be anticipated to reduce inflammation. Previous studies have shown that endothelium-derived NO is a potent anti-inflammatory molecule, suppressing the expression of chemokines and adhesion molecules mediating immune cell infiltration [49]. In this regard, there are data indicating that the number of immune cells in adipose tissue is related to insulin resistance [50]. Alternatively, it could be that reductions in ADMA and increased NOS activity could have a direct effect on adipose gene expression. In this respect we observed intriguing differences between the DDAH and eNOS-/- mice in adipose gene expression.

### NOS activity and adipose gene expression in response to diet

As shown in Table 2 the expression of markers characteristic for differentiated adipocytes, such as *Fabp4*, *Ucp1*, *Lpl *or *Lipe*, were downregulated in DDAH animals (ie. with higher NO bioavailability). By contrast, we observed upregulation of genes associated with lipogenesis in eNOS deficient mice. In the adipose tissue of these animals genes regulating adipogenesis (*Cebpa, Cebpb, Ppadb, Foxo1, Nr2f1, Mef2d, Ncor, Tgfb, Gdf10, Ucp1, Glut4) *as well as fatty acid *(Fasn, Acly) *and triglyceride *(Dgat2, Agpat2) *synthesis were upregulated. By comparison to the controls, genes concerning fatty acid oxidation were downregulated in both groups. The studies suggest that increased NO availability promotes changes in adipocyte gene expression, but not in adipogenesis. This may explain why, despite greater angiogenic capacity, the DDAH transgenic mice do not have greater adipogenesis.

Our microarray analysis of gene expression in WAT revealed that a number of genes involved in protection from oxidative stress such as *Fos*, *So d *or *Gstt *were upregulated in DDAH mice. This might reflect a compensatory response to nitrosative stress that may be induced by increased concentrations of the free radical NO [51]. Alternatively, this could reflect direct effects on gene expression by NO, as cGMP can increase the expression of superoxide dismutase 2 [52].

The main purpose of adipose tissue (especially WAT) is storage of high energy compounds [53]. In addition to regulating vascular tone, nitric oxide plays a crucial role in regulating metabolism of this tissue. It has been shown that in adipocytes, hepatocytes and myocytes nitric oxide activates glucose uptake as well as mitochondrial-biogenesis and catabolism (mitochondrial oxidation of glucose and FFA) [7]. These effects are observed in response to physiological concentrations of NO, endogenously produced by nNOS and eNOS [54]. These studies suggest a catabolic role for NO. The recent discovered mitochondrial NOS (mtNOS) may also play a role, as it maintains a low level of NO generation in healthy tissue (0.004-4.0 nmol NO min^-1 ^mg^-1 ^protein). The mtNOS has significant effects on cellular turnover, metabolism and survival [11,12].

## Conclusions

In summary, we conclude that changes in endogenous NOS activity alter the metabolic and genetic response to a high fat diet. An increase in endogenous eNOS activity (as in the DDAH mouse) is associated with an attenuation or reversal of fat-induced changes in blood sugar, adipocytokine levels, and adipogenesis gene expression. By contrast, a reduction in eNOS activity is associated with an increased susceptibility to fat-induced changes in gene expression that promote adipogenesis.

## List of Abbreviations

ADMA: asymmetric dimethylarginine; AMPK: 5' adenosine monophosphate-activated protein kinase; BAT: brown adipose tissue; bFGF: basic fibroblast growth factor; cGMP: cyclic guanosine monophosphate; DDAH: dimethylarginine dimethylaminohydrolase; eNOS-/- mice: endothelial nitric oxide deficient mice; FFA: free fatty acids; HFD: high fat diet; HGF: hepatocyte growth factor; Hsp90: heat shock protein 90; IGF-1: insulin - like growth factor; IL-6: interleukin 6; KO: knockout; L-NAME: NG-nitro-L-arginine methyl ester; NO: nitric oxide; NOS: nitric oxide synthase; PECAM-1 (CD31): platelet-endothelial cell adhesion molecule 1; PPARα: peroxisome proliferator-activated receptor α; TNF-α: tumor necrosis factor α; VEGF: vascular endothelial growth factor; WAT: white adipose tissue; WT mice: wild type mice.

## Competing interests

Dr. Cooke is the inventor of patents owned by Stanford University for diagnostic and therapeutic applications of the NOS pathway from which he receives royalties.

Other authors declare that they have no competing interests.

## Authors' contributions

UR drafted the manuscript, analysed data and participated in experiments in vivo. BKW participated in the drafting of the manuscript, design of the study and analysis of the data. LW helped to write the manuscript, participated in experiments in vivo, analysed the data and performed the statistical analysis. AP participated in the design of the study and carried out the molecular genetic studies. GD carried out and analysed immunohistology assays. BS helped to draft the manuscript. MM participated in the design of the study. RT participated in the design of the study and analysis of the data. JC helped to draft and edit the manuscript. ADK designed and coordinated the study as well as helped to draft the manuscript.

All authors read and approved the final manuscript.
